# Physical, mechanical, chemical and biological properties data of gellan gum incorporating titanium dioxide nanoparticles biofilm

**DOI:** 10.1016/j.dib.2020.105478

**Published:** 2020-04-08

**Authors:** Mohd Hasmizam Razali, Nur Arifah Ismail, Khairul Anuar Mat Amin

**Affiliations:** Faculty of Science and Marine Environment, Universiti Malaysia Terengganu, 21030 Kuala Nerus, Terengganu, Malaysia

**Keywords:** Physical, Chemical, Mechanical, Biological, Gellan gum, Titanium dioxide, Nanoparticles

## Abstract

Gellan gum incorporating titanium dioxide nanoparticles biofilm was synthesized and characterized using UV, FTIR and XRD to study their physical and chemical properties. The mechanical properties were measured using universal mechanical testing. Meanwhile, the biological properties were investigated towards for antibacterial and cell proliferation. This comprehensive data are relevant with the research article entitled “Gellan gum incorporating titanium dioxide nanoparticles biofilm as wound dressing: Physicochemical, mechanical, antibacterial properties and wound healing studies” [1].

Specifications table

**Table:** 

Subject	Chemistry, material science
Specific subject area	Synthesis and characterization of materials
Type of data	Table Image Graph Figure
How data were acquired	Data were acquired by UV–Vis, FTIR, XRD, universal testing machine, microscope
Data format	Raw Analyzed
Parameters for data collection	UV–Vis, FTIR and XRD were collected at room temperature. Universal testing machine was carried out according to the ASTM D882 [2]. Antibacterial activity of biofilms against bacteria was carried using disk diffusion after 24 h incubation. Meanwhile, cell viability and cell proliferation was measured after 24 h, 48 h, and 72 h incubation
Description of data collection	UV–Vis spectroscopy and FTIR spectra was scanned from 200 to 800 nm and 400 to 4000 cm^−1^, respectively. XRD diffractogram was performed by using Rigaku Miniflex (II) X-ray diffractometer operating at a scanning rate of 2.00° min^−1^, from 10° to 80° of 2θ at room temperature. Instron Universal Testing machine (model 3366) was used to study the mechanical properties of biofilms. The antibacterial testing was carried against Staphylococcus Aureus and Escherichia Coli bacteria strains. The viability of cells in contact with the biofilm samples for 24 h, 48 h, and 72 h incubation time was examined through a staining procedure of acridine orange/propidium iodide (AO/PI, Sigma Aldrich, USA) and observed by light microscope (Olympus IX73-FL-CCD). Meanwhile, the cells proliferations were quantified using a MTT (3-(4,5-dimethylthiazol-2-yl)−2,5-diphenyltetrazolium bromide) (Thermo Fisher Scientific, USA).
Data source location	Universiti Malaysia Terengganu Kuala Nerus/Terengganu Malaysia
Data accessibility	The data were found only in this article
Related research article	Ismail, N.A., Amin, K.A.M., Majid, F.A.A. and Razali, M.H., 2019. Gellan gum incorporating titanium dioxide nanoparticles biofilm as wound dressing: Physicochemical, mechanical, antibacterial properties and wound healing studies. *Materials Science and Engineering: C, 103*, p.109770. https://doi.org/10.1016/j.msec.2019.109770

## Value of the data

• Data obtained were important to know the physical, chemical, mechanical and biological properties of biofilm.
• Data may be useful for future research on the development of new nano-biocomposite film.
• These data were supported the good performance of fabricated biofilm for antibacterial and cell proliferation.

## 1 Data description

**Fig. 1:**
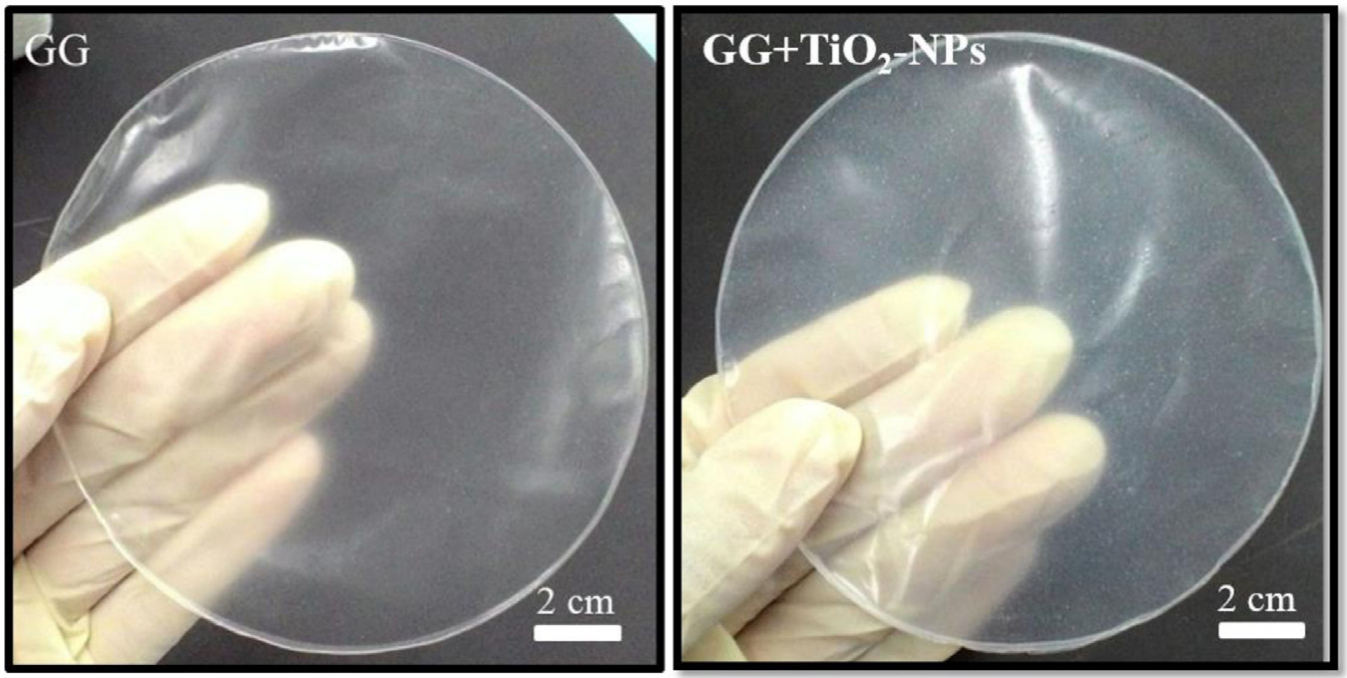
Photo images of GG and GG+TiO_2_-NPs biofilms.

**Fig. 2:**
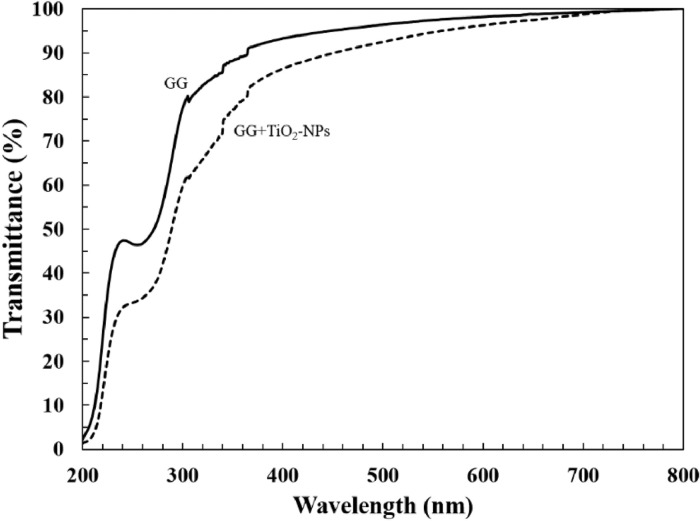
UV–Vis spectra of GG and GG+TiO_2_-NPs biofilms.

**Fig. 3:**
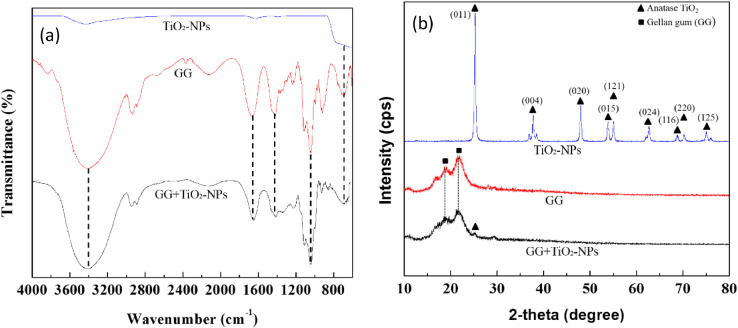
(a) FTIR spectra and (b) XRD diffractogram patterns of TiO_2_-NPs, GG and GG+TiO_2_-NPs biofilms.

**Fig. 4:**
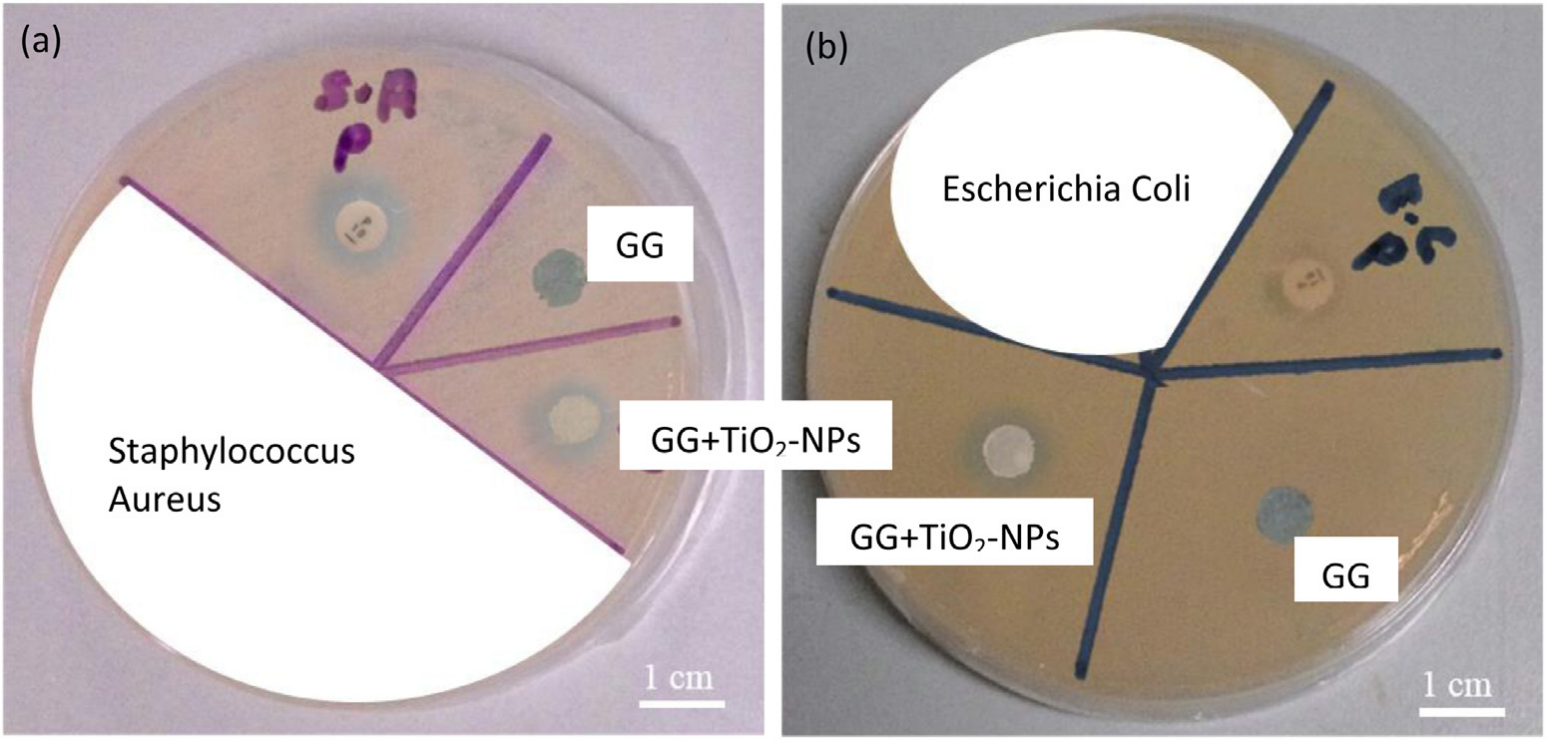
Photo images of antibacterial test result of penicillin (P), GG and GG+TiO_2_-NPs biofilms against (a) Staphylococcus Aureus and (b) Escherichia Coli bacteria.

**Fig. 5:**
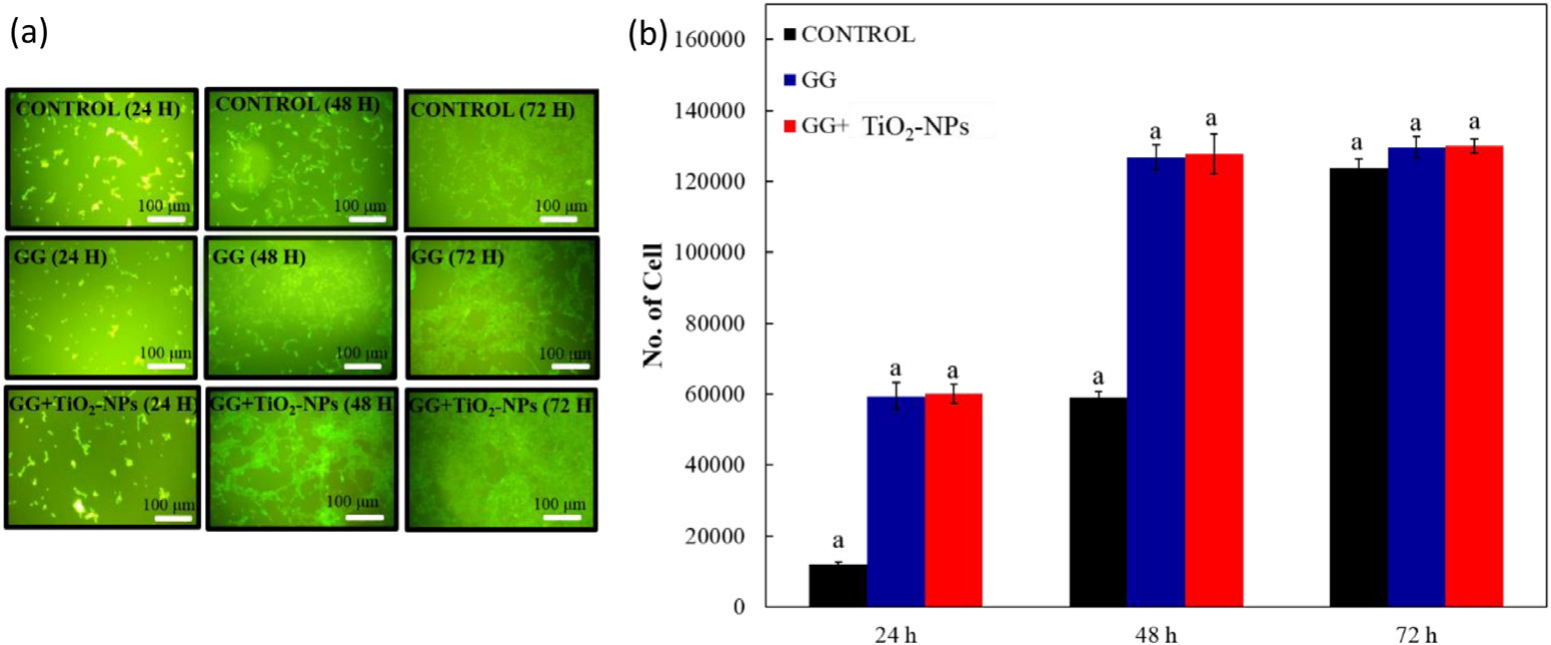
(a) Fluorescence microscope images of cell viability and (b) cell proliferation using control, GG, and GG+TiO_2_-NPs biofilms after 24, 48, and 72 h.

The dataset of this article provides information on physical, mechanical, chemical and biological properties of gellan gum incorporating titanium dioxide nanoparticles biofilms. Fig. 1 shows the photo images of pure gellan gum (GG) and gellan gum incorporating TiO_2_ nanoparticles (GG+TiO_2_-NPs) biofilms and their UV–Vis spectra were shown in Fig. 2. The mechanical properties of GG and GG+TiO2-NPs biofilms are presented in Table 1. Fig. 3(a) and (b) displays the FTIR spectra and XRD patterns of GG, and GG+TiO2-NPs biofilms as well as TiO_2_ nanoparticles (TiO_2_-NPs) powder. Photo images of antibacterial test against gram-positive (Staphylococcus Aureus*)* and gram-negative (Escherichia Coli*)* bacteria strains using pure GG, GG+TiO_2_-NPs biofilms and penicillin as a control sample were shown in Fig. 4(a) and (b), respectively. Their antibacterial activity was summarized in Table 2. Fig. 5(a) and (b) shows the fluorescence images of the cell viability and cell proliferation, respectively at different time interval.

**Table 1:** Thickness and mechanical properties of GG and GG+TiO_2_-NPs biofilms.

Sample	Thickness(µm)	TS(MPa)	YM(MPa)	T(J g^−1^)	EAB(%)
GG	60 ± 0.003	3.29 ± 0.06	58 ± 2.74	0.20 ± 0.008	13.21 ± 0.58
GG+TiO_2_-NPs	60 ± 0.004	3.76 ± 0.11	67 ± 3.41	0.17 ± 0.004	11.51 ± 0.65

(mean ± SD) (*n* = 5); TS (tensile strength), YM (Young’ Modulus), T (toughness), EAB (elongation-at-break).

**Table 2:** Inhibition zone of biofilms against Staphylococcus Aureus and Escherichia Coli.

Diameter of Inhibition (mm)		
Sample	Staphylococcus Aureus	Escherichia Coli
Penicillin (P)	12 ± 0.06	10 ± 0.12
GG	–	–
GG+TiO_2_-NPs	9 ± 0.25	11 ± 0.06

## 2 Experimental design, materials, and methods

Gellan gum incorporating TiO_2_ nanoparticles biofilm was fabricated using solvent casting method as used previously by other researchers [3,4]. Firstly, gellan gum solution was prepared by dissolving 1 g gellan gum in 100 mL deionized water under continuous stirring for 2 h at 70 °C. Glycerol (50 w/w%, percentage weight relative to GG) and calcium chloride (CaCl_2_) (5 mM) was added into the solution. Then, 1 w/w% (percentage weight relative to GG) of TiO_2_ nanoparticles was added and stirred for 2 h. The solution was transfer to casting dish and was dried in oven for 24 h at 50 °C to produce spherical shape biofilm. All biofilms were preconditioned in a desiccator (27 °C, 50% relative humidity (RH) for at least 2 days prior to testing. Pure GG biofilm was prepared using similar procedure with the absence of TiO_2_ nanoparticles. The obtained samples were characterized using UV–Vis, FTIR, XRD for physical and chemical characterization. The mechanical properties of biofilms were measured using Instron universal testing machine (model 3366) according to ASTM D882 [2]. Five specimens with the size of 2.0 × 6.0 cm rectangular strips of biofilm samples were tested. The biological properties were studied for antibacterial and cell proliferation. Gram-positive (*Staphylococcus aureus*) and Gram-negative (*Escherichia coli*) microbes were used for an anti-bacterial assay. Meanwhile, the cells proliferations were quantified using a MTT (3-(4,5-dimethylthiazol-2-yl)−2,5-diphenyltetrazolium bromide) (Thermo Fisher Scientific, USA). The viability of cells in contact with the biofilm samples for 24 h, 48 h, and 72 h of incubation time was examined through a staining procedure of acridine orange/propidium iodide (AO/PI, Sigma Aldrich, USA) and observed by light microscope (Olympus IX73-FL-CCD). Control in this experiment is Dulbecco's Modified Eagle Medium (DMEM) culture media without the presence of film samples.

## Appendix Supplementary materials

Image, application 1

Image, application 2
